# Two new species of *Brueelia* Kéler, 1936 (Ischnocera, Philopteridae) parasitic on Neotropical trogons (Aves, Trogoniformes)

**DOI:** 10.3897/zookeys.128.1583

**Published:** 2011-09-09

**Authors:** Michel P. Valim, Jason D. Weckstein

**Affiliations:** Field Museum of Natural History, Zoology Department, 1400 South Lake Shore Drive, Chicago, IL 60605, USA

**Keywords:** Chewing lice, *Brueelia*, trogons, Philopteridae, new species

## Abstract

Two new species of *Brueelia* are described and illustrated. These new species and their type hosts are: *Brueelia sueta* ex *Pharomachrus pavoninus* (Spix, 1824), the Pavonine Quetzal and *Brueelia cicchinoi* ex *Trogon viridis* Linnaeus, the White-tailed Trogon. Both new species differ from the only *Brueelia* described on *Trogon mexicanus* by many morphological features, including those present in the male genitalia and female vulvar margin. Partial sequences of the mitochondrial cytochrome oxidase I (COI) gene for these two new species differ from one another by 13.6% uncorrected p-distance. Whereas *Brueelia cicchinoi* is only 0.3% divergent from previously published COI sequences identified as *Brueelia* sp. from the Mexican *Trogon melanocephalus* Gould, 1936 and *Trogon massena* Gould, 1938. We also found *Brueelia cicchinoi* on *Trogon melanurus*, *Trogon collaris* and *Pharomachrus pavoninus*. Thus *Brueelia cicchinoi* is found on multiple trogoniform hosts across an extremely large geographic distribution and has one of the largest number of host associations among *Brueelia* species.

## Introduction

According to Price et al. (2003), *Brueelia* Kéler, 1936 (Ischnocera) is the most speciose genus of parasitic lice within the Philopteridae (Phthiraptera), with about 280 species currently described. Members of this genus parasitize the largest avian order, Passeriformes, but they also occur on Piciformes and Coraciiformes. Thus, due to the extremely large number of potential hosts, worldwide distribution, and complicated taxonomy there are probably hundreds of undescribed *Brueelia* species waiting to be discovered and described.

The only *Brueelia* species parasitic on trogons (Aves, Trogoniformes), *Brueelia insolita*, was described by Cicchino (1983) from *Trogon mexicanus* Swainson, 1827 collected in Guatemala, and this taxon was transferred to *Trogoninirmus* Eichler, 1944 by Price et al. (2003) without justification. Perhaps the authors simply assumed that trogons could not harbor species of *Brueelia*. However, the original description of *Brueelia insolita* is indubitably a *Brueelia* species (Cicchino 1983: 284) rather than a *Trogoninirmus* (Price et al., 2003: 245). A molecular phylogenetic study published by Johnson et. al. (2002) includes *Brueelia* specimens collected from two *Trogon* species and those specimens are genetically differentiated from all other *Brueelia* included in the study but fall phylogenetically within *Brueelia* parasitizing Passeriformes. This paper describes two additional species of *Brueelia* collected from Neotropical trogons.

## Methods

Specimens used in this study have been collected by the junior author and/or his colleagues using the Ethyl Acetate fumigation technique as described in Bueter et al (2009) and were mounted on slides following the procedures of Palma (1978). Nomenclature of the abdominal setae, different somatic features and abbreviations for the body measurements (given in millimeters) follow those proposed by Cicchino and Castro (1996). The abbreviations used were: HL, head length at midline; PAW, preantennal width; TW, temple width; PL, prothorax length; PW, prothorax width; PTL, pterothorax length; PTW, pterothorax width; AL, abdominal length; AW abdominal width (taken at segment V); GL, male genitalia length; and TL, total length. Host names were standardized following Dickinson (2003).

Using laboratory methods described by Bueter et al. (2009) we sequenced a 382 base pair (bp) portion of the mitochondrial cytochrome oxidase I (COI) gene from each of the new *Brueelia* species, including one *Brueelia sueta* sp. n. from *Pharomachrus pavoninus* and three individuals of *Brueelia cicchinoi* sp. n. from three different host species (*Trogon viridis*, *Trogon melanurus*, and *Pharomachrus pavoninus*) to assess and document their genetic distinctiveness. We made one minor modification to the voucher DNA extraction protocol. Rather than completely remove the louse head from the body for proteinase K digestion, we used a sterilized syringe needle to only partially cut the head from the body. This modification produced DNA vouchers better suited for morphological analysis and also minimized the chances of losing the head during extraction and slide mounting. The DNA sequences and their associated DNA voucher number are deposited in GenBank (JN384116-JN384119). We also incorporated the two COI sequences from *Brueelia* sp. collected from *Trogon massena* (AY149386) and *Trogon melanocephalus* (AY149387), from México published by Johnson et al. (2002) and put all of these trogon *Brueelia* COI sequences into a phylogenetic context using COI sequences from Bueter et al. (2009).

We used PAUP* (version 4.0b10; Swofford 2003) to calculate uncorrected p-distances between trogon *Brueelia* sequences and to conduct a maximum parsimony (MP) heuristic search and MP bootstrap analysis of the combined Bueter et al. (2009) and trogon *Brueelia* COI dataset. We conducted a MP heuristic search with TBR branch swapping, stepwise addition, and 100 random addition replicates. For the MP bootstrap analysis we performed 1000 bootstrap replicates with one random addition per replicate.

Holotypes of the new species are deposited in the Museu de Zoologia, University of São Paulo, São Paulo, Brazil (MZUSP) and paratypes are deposited in both MZUSP and the Field Museum of Natural History, Chicago, USA (FMNH). Other specimens studied are held in the Price Institute of Phthirapteran Research, University of Utah, Salt Lake City, USA (PIPeR). For material collected in 2005 and 2007 host specimen vouchers are deposited in the Museu Paraense Emilío Goeldi (MPEG) and FMNH and are indicated by field numbers and specimen numbers.

## Taxonomic treatment

### Brueelia Kéler, 1936

Type species *Brueelia rossittensis* Kéler, 1936 = *Brueelia brachythorax* (Giebel, 1874).

Type host: *Bombycilla garrulus* (Linnaeus, 1758) (Passeriformes, Bombycillidae).

#### Brueelia sueta

Valim & Weckstein sp. n.

urn:lsid:zoobank.org:act:476AC361-B912-4BDC-8373-483E37D76E49

http://species-id.net/wiki/Brueelia_sueta

Figs 1–6 13–14

##### Type host:

*Pharomachrus pavoninus* (Spix, 1824) – Pavonine Quetzal

##### Diagnosis.

This species is unique in the thickness of the temporal carina (Fig. 3) and by the shape of the anterior ventral plate (Figs 13–14) in both sexes. It is morphologically close to *Brueelia insolita* due the absence of postspiracular setae on segment IV in females, but they differ significantly in characters such as shape of the vulvar margin (with a notch in *Brueelia insolita*); number of setae on gonapophysis (six in *Brueelia insolita*), and more spiniform setae on vulvar margin. The males of both species can be distinguished by the shape of genitalia and the tendency to have two setae postspiracular accessories on tergites V–VIII (whilst *Brueelia insolita* has only one).

##### Male.

**Figures 1–6.**
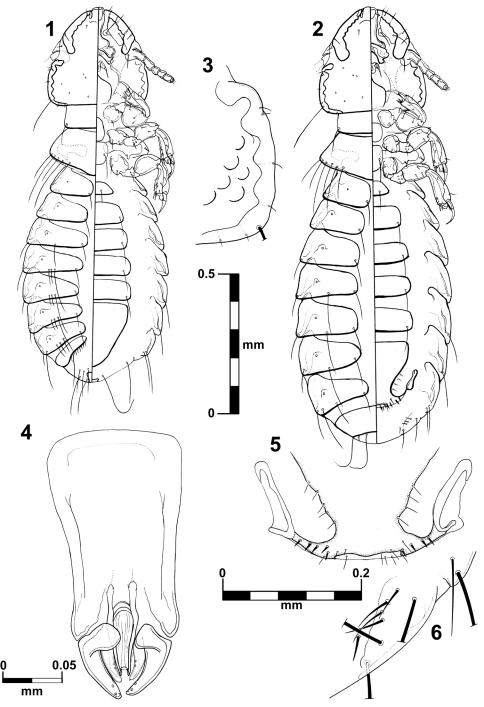
*Brueelia sueta* sp. n.: male, dorso-ventral views (**1**); female, dorso-ventral views (**2**); temporal carina (**3**); male genitalia (**4**); female vulvar margin (**5**); female gonapophysis (**6**).

Habitus as in fig. 1. Body pigmentation uniform, all plates barely yellowish slightly more pigmented on some details of the pleural areas. Head oval shaped, as long as wide. Small hyaline margin distinguishable; anterior dorsal head plate not completely surrounded by the dorsal preantennal suture. Preantennal margin slightly convex; marginal carina thickened with its inner margin sinuate, and completely pigmented (Figs 13–14). Tracks of cybarial muscles practically indistinct. Frontoclypeal suture with its nodal area well defined. Tracks of insertion of the mandibular adductor muscles well marked. Gular plate well pigmented with a broad rhombic silhouette. Temples forming an acute angle at level of the marginal temporal setae 3; temporal carina pigmented and thick, with its inner margin deeply sinuate (Fig. 3); eye imbedded within thickened carina making its distinction on margin of the head difficult (Fig. 3). Pterothorax with 5–7 marginal setae on each side; pterothoracic apodeme well developed, not reaching the lateral margin of the pterothorax. Mesosternal and metasternal plates not fused, both slightly longer than wide, only the metasternal plate bearing two long setae. Abdomen with tergites II–VIII lightly and uniformly pigmented. Tergal chaetotaxy: postspiracular long on IV–VIII; two small accessory setae on V–VIII (atypical specimens with only one seta in one side); and one sutural seta on II–VIII. Tergite IX+X (from the lateral to meson) with one short, one long and six (rarely seven) short setae. Paratergal chaetotaxy: II–III 0; IV–V 1; VI–VII 2; VIII 4. Sternal plates II–VI yellowish, typically with one pair of setae on each, subgenital plate uniformly pigmented. Genitalia (Fig. 4): basal plate wide, with sub-parallel lateral borders; straight and broad subtriangular paramera, with rhombic tips (Fig. 4); lateral sclerites of the endomeral complex long (2/3 of the paramera length) subtriangular with their posterior edge smooth, bearing 2 sensillae each.

*Body measurements* (n = 4): HL, 0.33–0.35; PAW, 0.27–0.28; TW, 0.35–0.36; PL, 0.13–0.14; PW, 0.24–0.25; PTL, 0.15–0.16; PTW, 0.34–0.35; AL, 0.71–0.80; AW 0.48–0.52; GL, 0.23; and TL, 1.25–1.35.

##### Female.

Habitus as in figure 2. Pigmentation of the head, thorax and abdomen much as for male, differing in body size, terminalia and tergal chaetotaxy (one long postspiracular seta on V–VIII). Pterothorax with 4–6 marginal setae on each side. Tergites II–VIII divided medially, IX+X entire and uniformly pigmented. Subgenital plate uniformly pigmented, lacking posterior notch, with 3–5 small setae each side (Fig. 5). Gonapophysis commonly with 4 setae (Figs 6). Vulva with 4–5 short and spiniform setae, and 3–6 (rarely 2) long and thin setae on each side (Fig. 5).

*Body measurements* (n = 4): HL, 0.37–0.38; PAW, 0.30–0.31; TW, 0.38–0.39; PL, 0.13–0.16; PW, 0.26–0.27; PTL, 0.14–0.16; PTW, 0.37–0.38; AL, 0.94–1.10; AW 0.50–0.59; and TL, 1.52–1.67.

##### Type material.

Male holotype, ex *Pharomachrus pavoninus*, JAP766 MPEG 62493; BRAZIL: Amazonas, Maraã, Lago Cumapi (01°43'48.6"S; 65°52'45.5"W ), 31.VII.2007, J.D. Weckstein col., at MZUSP. Paratypes: 3 males and 4 females (one female DNA voucher Brsp.Phpa.1.4.2011.19), same data as holotype. 1 male and 1 female (DNA voucher) paratypes at FMNH.

##### Etymology.

The epithet derives from *suetus* (L.), which means: wont; accustomed; usual. It makes reference to the fact of the genus *Brueelia* is a common parasite on trogons, rather than *insolitus* (L., unusual) as believed by Cicchino (1983) who described one species of this louse genus from this host group.

#### Brueelia cicchinoi

Valim & Weckstein sp. n.

urn:lsid:zoobank.org:act:5EF8725E-FAE3-4B43-B4F0-2EC82E090B62

http://species-id.net/wiki/Brueelia_cicchinoi

Figs 7–12 15–16

##### Type host:

*Trogon viridis* Linnaeus, 1766 – White-tailed Trogon

##### Diagnosis.

The shape of the anterior ventral plate is unique in this species (Figs 15–16). It is morphologically close to *Brueelia insolita* by the thickness of the temporal carina and by the presence of setae on the mesosternal plate; but they can be distinguished by the shape of the anterior ventral plate. The males of this species can be distinguished by the distinct genital architecture and by the presence of two long setae on tergite IX+X (only one in *Brueelia insolita* and *Brueelia sueta* sp. n.). In females, the presence of postspiracular setae on segment IV; the chaetotaxy of vulvar margin; and lacking of the notch on vulvar margin are the most distinctive characters. This species can be promptly distinguished from the *Brueelia sueta* sp. n. by the thickness of the temporal carina, genitalia and tergal chaetotaxy in males; and by the presence of postspiracular setae on tergite IV and vulvar chaetotaxy in females.

##### Male.

**Figures 7–12.**
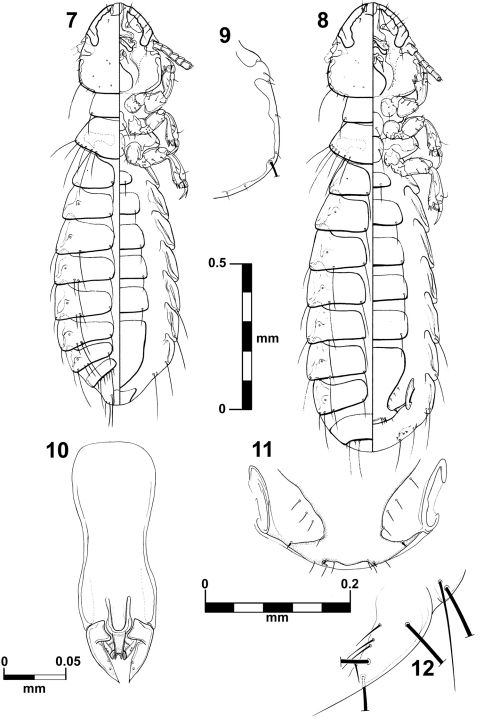
*Brueelia cicchinoi* sp. n.: male, dorso-ventral views (**7**); female, dorso-ventral views (**8**); temporal carina (**9**); male genitalia (**10**); female vulvar margin (**11**); female gonapophysis (**12**).

Habitus as in fig. 7. Body pigmentation uniform, all plates barely yellowish in color. Head oval shaped, slightly longer than wide. Hyaline margin indistinguishable; anterior dorsal head plate not completely surrounded by the dorsal preantennal suture. Preantennal margin slightly convex; marginal carina thickened with its inner margin sinuate (Figs 15–16). Tracks of cybarial muscles practically indistinct. Frontoclypeal suture with its nodal area well defined. Tracks of insertion of the mandibular adductor muscles faintly marked. Gular plate well pigmented with a broad rhombic silhouette. Temples more rounded; temporal carina pigmented and thinner, with its inner margin only superficially sinuate (Fig. 9); eye distinct from the temporal carina (Fig. 9). Pterothorax with 5–6 marginal setae on each side; pterothoracic apodeme well developed, not reaching the lateral margin of the pterothorax. Mesosternal and metasternal plates not fused, both slightly longer than wide and bearing two long setae each. Abdomen with tergites II–VIII lightly and uniformly pigmented. Tergal chaetotaxy: postspiracular long on IV–VIII; one small accessory setae on V–VIII (atypical specimens lack this seta on one side); and one sutural seta on II–VIII. Tergite IX+X (from the lateral to meson) with one short, one long, three short, one long, and one short setae. Paratergal chaetotaxy: II–III 0; IV–V 1; VI–VII 2; VIII 3. Sternal plates II–VI yellowish, typically with one pair of setae on each, subgenital plate uniformly pigmented. Genitalia (Fig. 10): basal plate wide, with concavity on lateral borders; straight and broad subtriangular paramera, with pointed tips (Fig. 10); lateral sclerites of the endomeral complex short (1/3 of the paramera length) and subtriangular with their posterior edge smooth, bearing 2 sensillae each.

*Body measurements* (n = 5), ex *Trogon viridis*: HL, 0.30–0.31; PAW, 0.23–0.24; TW, 0.28–0.30; PL, 0.11–0.14; PW, 0.20; PTL, 0.12–0.16; PTW, 0.27–0.28; AL, 0.77–0.87; AW 0.37–0.42; GL, 0.19–0.21; and TL, 1.27–1.38.

*Body measurements* (n = 3), ex *Trogon massena*: HL, 0.31–0.33; PAW, 0.24–0.25; TW, 0.29–0.30; PL, 0.12–0.13; PW, 0.20–0.21; PTL, 0.13; PTW, 0.29–0.31; AL, 0.83–0.93; AW 0.39–0.47; GL, 0.20–0.21; and TL, 1.34–1.48.

*Body measurements* (n = 1), ex *Trogon melanocephalus*: HL, 0.33; PAW, 0.26; TW, 0.31; PL, 0.13; PW, 0.21; PTL, 0.14; PTW, 0.32; AL, 0.91; AW 0.50; GL, 0.19; and TL, 1.46.

*Body measurements* (n = 2), ex *Trogon collaris*: HL, 0.30–0.31; PAW, 0.23–0.24; TW, 0.29–0.30; PL, 0.11–0.13; PW, 0.19–0.21; PTL, 0.15; PTW, 0.28–0.30; AL, 0.83–0.84; AW 0.39–0.43; GL, 0.18–0.20; and TL, 1.37–1.38.

##### Female.

Habitus as in figure 8. Pigmentation of the head, thorax and abdomen much as for male, differing in body size, terminalia and tergal chaetotaxy. Pterothorax with 5–6 marginal setae on each side. Tergites II–VIII divided medially, IX+X entire and uniformly pigmented. Subgenital plate uniformly pigmented, lacking posterior notch, with 3–4 small setae each side (Fig. 11). Gonapophysis commonly with 3 setae (Figs 12). Vulva with 2–3 short and spiniform setae and 2–3 long and thin setae on each side (Fig. 11).

*Body measurements* (n = 3), ex *Trogon viridis*: HL, 0.32–0.33; PAW, 0.26; TW, 0.32; PL, 0.12–0.13; PW, 0.21–0.22; PTL, 0.13–0.15; PTW, 0.30–0.31; AL, 0.98–1.07; AW 0.44–0.46; and TL, 1.53–1.61.

*Body measurements* (n = 2), ex *Trogon melanurus*: HL, 0.34–0.36; PAW, 0.26–0.27; TW, 0.33–0.34; PL, 0.15–0.16; PW, 0.23; PTL, 0.15; PTW, 0.33; AL, 0.98–1.05; AW 0.46–0.47; and TL, 1.56–1.60.

*Body measurements* (n = 3), ex *Trogon massena*: HL, 0.34; PAW, 0.26–0.28; TW, 0.32–0.33; PL, 0.13; PW, 0.21–0.23; PTL, 0.15–0.16; PTW, 0.32–0.33; AL, 1.01–1.10; AW 0.46–0.47; and TL, 1.59–1.69.

*Body measurements* (n = 1), ex *Trogon melanocephalus*: HL, 0.35; PAW, 0.27; TW, 0.33; PL, 0.14; PW, 0.22; PTL, 0.14; PTW, 0.34; AL, 1.09; AW 0.49; and TL, 1.68.

*Body measurements* (n = 3), ex *Trogon collaris*: HL, 0.33–0.34; PAW, 0.26–0.27; TW, 0.32–0.33; PL, 0.13; PW, 0.20–0.22; PTL, 0.14–0.17; PTW, 0.32–0.33; AL, 1.01–1.08; AW 0.48–0.54; and TL, 1.58–1.66.

##### Type material.

Male holotype, ex *Trogon viridis*, JAP765 FMNH 456563; BRAZIL: Amazonas, Maraã, Lago Cumapi (01°43'48.6"S; 65°52'45.5"W ), 31.VII.2007, J.D. Weckstein col., at MZUSP. Paratypes: 4 males and 3 females (one female DNA voucher Brsp.Trvi.1.4.2011.20), same data as holotype. 2 males and 1 female (DNA voucher) paratypes at FMNH.

**Figures 13–16.**
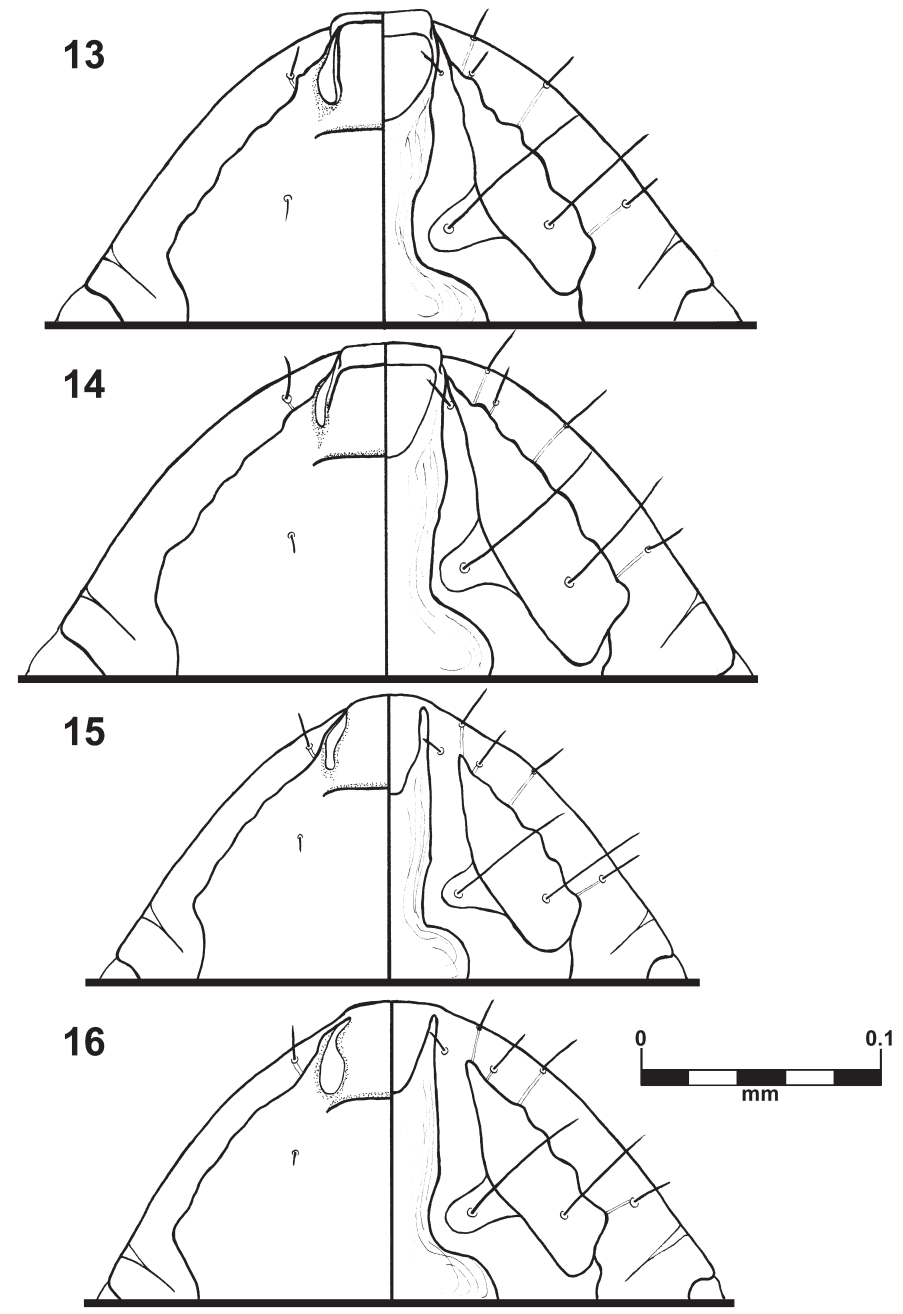
*Brueelia sueta* sp. n.: male preantenal region, dorso-ventral views (**13**); female preantenal region, dorso-ventral views (**14**); *Brueelia cicchinoi* sp. n.: male preantenal region, dorso-ventral views (**15**); female preantenal region, dorso-ventral views (**16**).

##### Other specimens studied not regarded as types.

1 male and 1 female (female DNA voucher Brsp.Phpa.4.4.2011.16), ex *Pharomachrus pavoninus*, JAP315 MPEG 62491; BRAZIL: Amazonas, Japurá, Rio Mapari (02°02'31.5"S; 67°17'16.6"W ), 17.VII.2007, J.D. Weckstein col., at FMNH; 2 females (one female DNA voucher Brsp.Trme.4.4.2011.13), ex *Trogon melanurus*, AMZ415 MPEG 59344; BRAZIL: Amazonas, Barcelos, Rio Aracá (0°25'12"S; 62°56'13"W ), 4.XII.2005, C.C. Ribas col., at MZUSP; 4 males and 4 females (one pair of specimens DNA vouchers Trsp.Trmas.3.29.1999.4 and Trsp.Trmas.4.7.1999.9), ex *Trogon massena*; MÉXICO: Campeche, 24 km S Sivituc (18°14'N; 90°12'W ), 6.III.1998, D.H. Clayton col., at PIPeR; 1 male and 2 females (one female DNA voucher Trsp.Trmel.5.4.1999.5); ex *Trogon melanocephalus*; MÉXICO: Campeche, 24 km S Sivituc (18°14'N; 90°12'W ), 9.III.1998, D.H. Clayton col., at PIPeR; 2 males and 3 females (FMNH-INS 28922, 28923); ex *Trogon collaris*; PERU: Madre de Dios, Hacienda Amazonia, near Atalaya ridged, 330m above hacienda, 5.VIII.1985, D.H. Clayton col. (#85-076 and 85-077), at FMNH.

##### Etymology.

This species is named after Armando C. Cicchino (Universidad Nacional de Mar del Plata, Mar del Plata, Argentina) in recognition of his more than thirty years of contributions to the taxonomy and systematics of the genus *Brueelia* and chewing lice in general.

##### Remarks.

Although we described herein a new *Brueelia* species from *Pharomachrus pavoninus*, this same host species, from a different locality, also harbored *Brueelia cicchinoi* sp. n. Nevertheless, we are certain that this record of *Brueelia cicchinoi* on the host *Pharomachrus pavoninus* is a reliable host-parasite association because: (1) the pair of *Brueelia* specimens collected from *Pharomachrus pavoninus* are morphologically identical with those collected from *Trogon viridis*; (2) both individuals of *Pharomachrus pavoninus* were collected on different days and at different localities; (3) no *Trogon* spp. were deloused or collected on the day that JDW collected *Brueelia cicchinoi* sp. n. from *Pharomachrus pavoninus*; (4) specimens of *Brueelia cicchinoi* sp. n. collected from *Pharomachrus pavoninus* and *Trogon viridis* are genetically identical (see below); (5) our specimens are only 1 bp different and thus nearly genetically identical to *Brueelia* sp. collected from two other *Trogon* species (see below) collected in México. Lastly, although the type host of *Brueelia cicchinoi* sp. n., *Trogon viridis* MPEG 62484, was collected in the Amazonian Imerí area of endemism between the Rio Japurá and Rio Negro, we also found *Brueelia cicchinoi* sp. n. on *Trogon melanurus* MPEG 59344) from the Imerí area of endemism between the Rio Branco and the Rio Negro and on *Pharomachrus pavoninus* MPEG 62491 from the Napo area of endemism south of the Rio Japurá. Although we do not have a male specimen of *Brueelia cicchinoi* sp. n. from *Trogon melanurus* we are also certain of this host association because the two specimens studied from *Trogon melanurus* are morphologically and genetically indistinguishable from those collected from *Trogon viridis*. Thus, *Brueelia cicchinoi* sp. n. is apparently a relatively widespread trogon parasite found on at least six species of trogons: *Trogon viridis*, *Trogon melanurus*, *Trogon collaris*, *Trogon massena* (see below), *Trogon melanocephalus* (see below), and *Pharomachrus pavoninus*.

#### Brueelia insolita

Cicchino, 1983

http://species-id.net/wiki/Brueelia_insolita

Brueelia insolita Cicchino, 1983: 284, Figs 7–13; Type host *Trogon mexicanus*; Soloma, Huehuetenango, GUATEMALA.
Trogoninirmus insolitus , Price et al. 2003: 245 (incorrectly included in *Trogoninirmus*).

##### Diagnosis.

This species is readily distinguished from the other species herein described by the size and shape of the male genitalia and the conspicuous central notch present on the vulvar margin in females. Its head carina and body chaetotaxy are close to that found on *Brueelia cicchinoi* sp. n.

##### Male body measurements

(n=1): HL, 0.34 (0.32); PAW, 0.28 (0.25); TW, 0.33 (0.31); PL, 0.13 (0.12); PW, 0.23 (0.21); PTL, 0.15; PTW, 0.34 (0.33); AL, 0.78 (0.70); AW 0.48 (0.46); GL, 0.26; and TL, 1.34 (1.26).

##### Female body measurements

(n=6): HL, 0.37–0.38 (0.34); PAW, 0.29–0.31 (0.27); TW, 0.36–0.37 (0.33); PL, 0.13–0.15 (0.12); PW, 0.23–0.25 (0.22); PTL, 0.15–0.18 (0.15); PTW, 0.34–0.36 (0.32); AL, 0.99–1.12 (0.94); AW 0.54–0.57 (0.52); and TL, 1.58–1.69 (1.54).

##### Specimens studied.

1 male and 6 females, ex *Trogon mexicanus*, DB703; GUATEMALA: Huehuetenango, Soloma, 12.IX.1958, D. Baepler col., at PIPeR.

##### Remarks.

Unexpectedly we had the opportunity to discover and analyze specimens from the same lot as those used by Cicchino (1983). As these additional specimens agree completely with the description provided by that author and his description is very precise for recognizing this taxon no redescription is necessary. However the type series of *Brueelia insolita* is based only on a pair of specimens, and thus here we present additional morphometric data for this newly analyzed material. The original measurements provided by Cicchino (1983) for his male *Brueelia insolita* were similar to the measurements of the specimens from PIPeR and his measurements of the female specimen fell within our measurements to two decimals. Cicchino’s original measurements are provided in parenthesis.

## Discussion

Based on a MP bootstrap analysis of the partial mitochondrial COI sequences, support for the monophyly of trogon *Brueelia* was only 56%. However, all 48 of the MP trees (TL=913, CI=0.36, RI=0.70) indicated that *Brueelia* parasitizing trogons were monophyletic. Furthermore, uncorrected p-distances indicate that the morphologically diagnosable *Brueelia* species, *Brueelia sueta* sp. n. and *Brueelia cicchinoi* sp. n., are differentiated genetically as well. Uncorrected p-distances between them range from 13.4–13.6%, whereas uncorrected p-distances within *Brueelia cicchinoi* sp. n. from the five different host taxa *Trogon viridis*, *Trogon melanurus*, *Trogon massena*, *Trogon melanocephalus*, and *Pharomachrus pavoninus* are extremely small and range from 0–0.3%. *Brueelia cicchinoi* sp. n. from Amazonian *Trogon viridis*, *Trogon melanurus*, and *Pharomachrus pavoninus* are identical across 382 bp of COI. However, these three specimens differ by a single bp from the Mexican *Brueelia* sp. from *Trogon massena* and *Trogon melanocephalus* sequenced by Johnson et al. (2002). Thus, the similarity of these COI sequences and the morphological features of the *Brueelia* sp. from *Trogon massena* and *Trogon melanocephalus* are consistent with these Mexican specimens being *Brueelia cicchinoi* sp. n. as well.

Our data in combination with data published by Johnson et al. (2002) suggests that *Brueelia cicchinoi* sp. n. is a relatively widespread trogon parasite. In addition to parasitizing at least six species of trogons including *Trogon viridis*, *Trogon melanurus*, *Trogon collaris*, *Trogon massena*, *Trogon melanocephalus*, and *Pharomachrus pavoninus*, *Brueelia cicchinoi* sp. n. is found from North America as far south as Departamento Madre de Dios, Peru and east to eastern Amazonas, Brazil and thus includes the Inambari, Napo, and Imerí Amazonian areas of endemism. This broad louse distribution is extraordinary because it crosses a number of major avian biogeographic barriers including the Andes. In general, the Trogonidae are not long distance migrants, although *Pharomachrus* is a known altitudinal migrant (Collar 2001) and genetic data indicates that widespread trogon taxa such as *Trogon viridis* are not crossing the Andes (Dacosta and Klicka 2008). Thus the lice are not being carried across these barriers by the birds. However, the distribution of Trogonidae is more or less continuous across the lowlands and highlands of Central and South America, which suggests that perhaps the lack of host specificity of *Brueelia cicchinoi* allows it to use a variety of trogon taxa that inhabit different elevations and habitat types (e.g. highlands and dry forest) as a bridge between geographic regions. This might explain the large geographic distribution of this parasite and lack of genetic divergence between individuals in México and Amazonia despite the host taxa having much more limited distributions. Additional collections across the Neotropics will help us to better understand this pattern and the potential process of parasites dispersing while their hosts are relatively sedentary.

## Supplementary Material

XML Treatment for Brueelia sueta

XML Treatment for Brueelia cicchinoi

XML Treatment for Brueelia insolita
